# Partial Bell-State Measurement with Type-II Parametric Down Conversion: Extracting Phase Information from the Zeropoint Field (I)

**DOI:** 10.3390/e25030393

**Published:** 2023-02-21

**Authors:** Alberto Casado, Santiago Guerra

**Affiliations:** 1 Departamento de Física Aplicada III, Escuela Técnica Superior de Ingeniería, Universidad de Sevilla, 41092 Sevilla, Spain; 2 Thermal Engineering & Instrumentation Division (IDeTIC), Universidad de Las Palmas de Gran Canaria, 35017 Las Palmas de Gran Canaria, Spain

**Keywords:** zeropoint field, Wigner representation, parametric down-conversion, entanglement, Bell-state analysis

## Abstract

In this paper, the nexus between the Bell-state measurement and extracting phase information from the zeropoint field is investigated. For this purpose, the Wigner representation in the Heisenberg picture is applied in a Bell-type experiment in which the polarisation-entangled photon pairs generated in a type-II parametric down-conversion do not overlap. The signal intensities at the detectors are calculated in a four-mode approximation, being expressed as functions of the modules and phases of the four zeropoint amplitudes entering the crystal. A general criterion for identifying the correlated detectors is proposed based on the equality of the signal intensities, and without involving the calculation of the joint detection probabilities. In addition, from the analyses in the rectilinear and diagonal basis, it is shown that the distinguishability of the polarisation Bell states, which is in direct correspondence with the joint detection events in each experiment, can be related to the knowledge of the phases of the vacuum field entering the entanglement source, and giving rise to correlated detections. To this purpose, it is conjectured that a detection event is associated with a maximum value of the signal intensity averaged in the modules of the zeropoint amplitudes, as a function of the vacuum phases.

## 1. Introduction

In the last few years, the role of the zeropoint field (ZPF) in optical quantum communication using a parametric down-conversion (PDC) has been investigated [1]. To achieve this goal, the Wigner representation in the Heisenberg picture (WRHP) has been used [2]. New physical insights allow for a profound understanding of the optical implementation of quantum information using PDC. Specifically, the use of the WRHP formalism in experiments on quantum cryptography [3], Bell-state analysis [4] and teleportation [5] has been revealed as a new perspective focused on the wave nature of light.

As described elsewhere [4], the zeropoint inputs that intervene in a given experiment on Bell-state measurement (BSM) of two photons entangled in *n* dichotomic degrees of freedom, using linear evolution and local measurement (LELM), are related to the maximal information that can be extracted in the experiment. On the one hand, the quantum information is storaged in the vacuum amplitudes that are amplified at the source of entanglement. On the other hand, the zeropoint inputs corresponding to the idle channels inside the Bell-state analyser constitute a source of noise that limits the capacity for extracting information. At a more concrete level, it has been demonstrated that the maximal distinguishability in experiments in which the two photons are not brought together at the apparatus is just the difference between the number of sets of zeropoint amplitudes that are amplified at the source and enter the analyser, and the corresponding one to the zeropoint inputs inside the area of a single detection event.

The question that arises naturally is whether the total information extracted via Bell-state analysis can be associated with some piece of information related to the sets of zeropoint amplitudes entering the source of entanglement. The following reasoning supports an affirmative response: given that the distinguishability of Bell-states is in direct correspondence with the number of zeropoint inputs at the crystal and the analyser, there should be a correspondence between the information concerning the distinguishable Bell states and the one corresponding to the values of the vacuum amplitudes that are amplified at the source. In this way, both formalisms, the Hilbert-space approach that highlights the particle behaviour of light through the concept of photon, and the WRHP formalism that resembles the undulatory behaviour of light through the consideration of the ZPF, would be in total correspondence. Hence, a given optical experiment in which a Bell state is measured should extract some information about the amplitudes of the zeropoint field at the crystal, concretely the vacuum phases. Needless to say, the amount of information, either concerning Bell states or vacuum phases, should be the same. To this purpose, the WRHP formalism is applied to a general Bell-type experiment in which the two photons, entangled in any of the four polarisation Bell states (n=1), do not overlap at the Bell-state analyser [6].

The paper is organised as follows. Section 2 and Section 3 contain introductory material. In Section 2, some basic aspects of the Bell-state analysis in the Hilbert-space formalism are reviewed. The relevant ingredients of the WRHP description of entanglement generated via type-II PDC, which are necessary for the understanding and notation of the rest of the paper, are given in Section 3. In Section 4, the field amplitudes at the detectors are calculated as functions of the experimental parameters and the modules and phases of the zeropoint amplitudes entering the crystal. The key contributions to this paper are given in Section 5, Section 6, Section 7 and Section 8. In Section 5, the relationship between the partial analysis of Bell states and zero point inputs is reviewed and extended to the complete analysis when two consecutive experiments, one of them in the rectilinear basis and the other in the diagonal one, are performed. In Section 6, the signal intensities at the detectors, i.e., the intensities above the zeropoint background are calculated and expressed as functions of the vacuum amplitudes and the experimental parameters. The rectilinear and diagonal analyses are described in Section 7. It is demonstrated that the signal intensities corresponding to the correlated detectors are exactly the same, so that a general criterion for establishing which detectors are correlated can be defined without the calculation of the joint detection probabilities. In this sense, this paper constitutes a general extension of the ideas developed in Section 3 of [1], where an analysis of this experiment in the rectilinear basis was made. In Section 8, in order to establish a correspondence between the which-path information and phase information, both leading to the distinguishability of Bell states, a conjecture is made concerning the relationship between a joint detection event in ideal photodetectors and the maximum value taken by the signal intensity averaged in the modules of the zeropoint amplitudes, as a function of the vacuum phases. As a consequence, a table of information is generated with a one-to-one correspondence between the values of the vacuum phases and the sequence of joint detection events for two copies of each of the four Bell states, when the two analyses are made consecutively. Finally, in Section 9, the main conclusions and further steps of this paper are presented.

## 2. General Aspects of BSM in the Hilbert Formalism

Type-II PDC is widely used as a source of entangled states in optical experiments for testing Bell inequalities [7,8,9,10,11,12] and the implementation of quantum communication schemes [13,14,15,16,17,18]. A monochromatic pumping laser impinges on one side of a nonlinear crystal, giving rise to pairs of conjugated beams with orthogonal polarisations. In the Hilbert formalism, the corpuscular nature of light is emphasised. In this approach, a photon of the laser field, with frequency $\omega_{0}$ and wave vector $k_{0}$, splits into two photons, 1 and 2, with frequencies $\omega_{1}$ and $\omega_{2}$, and wave vectors $k_{1}$ and $k_{2}$, fulfilling the exact matching conditions $\omega_{0}=\omega_{1}+\omega_{2}$ and $k_{0}=k_{1}+k_{2}$, which are related to energy and momentum conservation, respectively. If the two photons correspond to the intersection between the ordinary and extraordinary cones, the emitted state is entangled in polarisation [19]. Then, the manipulation of one of the particles via a unitary transformation gives rise to the generation of any of the four Bell states, which constitute a basis of the Hilbert space corresponding to the two particle system:

(1) $$|\Psi^{\pm }\rangle =\frac{1}{\sqrt{2}}\left[{|H\rangle }_{1}{|V\rangle }_{2}\pm {|V\rangle }_{1}{|H\rangle }_{2}\right],$$

(2) $$|\Phi^{\pm }\rangle =\frac{1}{\sqrt{2}}\left[{|H\rangle }_{1}{|H\rangle }_{2}\pm {|V\rangle }_{1}{|V\rangle }_{2}\right],$$

where *H* represents extraordinary, i.e., horizontal polarisation, and *V* represents ordinary, i.e., vertical polarisation. Equations (1) and (2) describe the entanglement between two particles (photons) that have been generated at the crystal, which propagate throughout the experimental setup and are finally detected.

The possibility of measuring the four Bell states is the base of quantum dense coding, consisting of the sending of two bits of classical information by only manipulating one of the qubits [14]. In addition, BSM is an essential ingredient in quantum teleportation [15]. Let us consider two photons entangled in *n* dichotomic degrees of freedom. The corresponding Hilbert-space is $4^{n}$ dimensional, and the standard analysis gives an upper bound to the maximal distinguishability of Bell-state classes equal to $2^{n+1}$, since the first detection event does not provide any information about the Bell state. The optimal BSM allows for the distinguishability of $N_{max,class}=2^{n+1}-1$ Bell-state classes when the two photons are overlapped at a balanced beam-splitter [20]. In contrast, if the two photons are not brought together at the apparatus, then the maximal distinguishability is given by $N_{max,class}=2^{n}$.

As it is well-known, in the case n=1, the four polarisation Bell states cannot be distinguished by using a LELM apparatus [21]. If the two photons are overlapped at a balanced beam-splitter, then $N_{max,class}=3$. If the two photons do not interact, only two classes can be distinguished. The use of hyperentanglement, i.e., the entanglement in n>1 degrees of freedom, allows for the BSM of the four polarisation Bell states [22,23,24].

**Figure 1:**
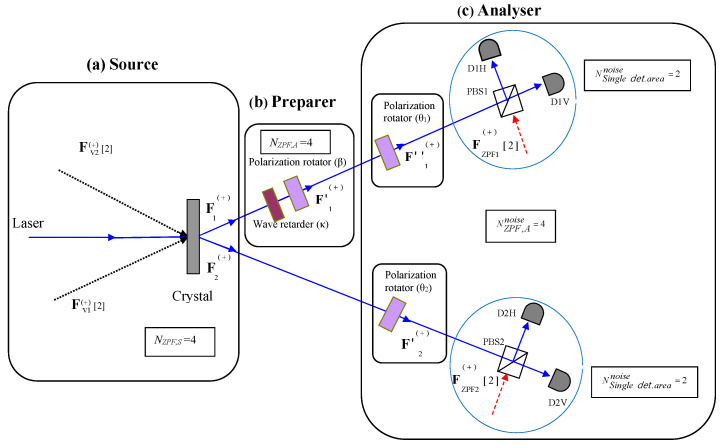
Setup for partial Bell-state analysis. The different zeropoint inputs are represented, and the number of sets of vacuum modes is written between brackets. (**a**) **Source**. Two correlated beams, $F_{1}^{\left(+\right)}$ and $F_{2}^{\left(+\right)}$, are generated via the coupling between the zeropoint field amplitudes, $F_{v1}^{\left(+\right)}$ and $F_{v2}^{\left(+\right)}$, and the laser beam into the crystal. The number of sets of ZPF modes that enter the source is ($N_{ZPF,S}=4$). (**b**) **Preparer**. Beam $F_{1}^{\left(+\right)}$ is modified using a wave retarder (κ) and a polarisation rotator (β). (**c**) **Analyser**. The polarisation of beam 1 (2) is analysed by using a polarisation rotator of angle $\theta_{1}$ ($\theta_{2}$) and a polarizing beam-splitter. Each of the idle channels of the PBSs introduces two sets of zeropoint modes, so that the number of sets of ZPF modes entering each single detection area is ($N_{Sing.det.area}^{noise}=2$). The maximum distinguishability corresponding to this setup is $N_{max.class}=N_{ZPF,S}-N_{Sing.det.area}^{noise}=4-2=2$, and it can be accomplished only in the cases of rectilinear ($\theta_{1}=\theta_{2}=0$) or diagonal ($\theta_{1}=\theta_{2}=\pi /4$) analyses.

In the experimental setup shown in Figure 1, photon 1 is manipulated via a wave retarder that introduces a phase shift κ={0,π} between the vertical and horizontal component of the electric field, and a polarisation rotator of angle β={0,π/2}. This allows for the generation of any of the four polarisation Bell states (see Equations (1) and (2)). For instance, if the emitted state is $|\Psi^{+}\rangle$, the values (κ=0,β=0) would correspond to the identity operation, and the pair (κ=π,β=0) would transform this state in $|\Psi^{-}\rangle$. On the other hand, (κ=0,β=π/2)[(κ=π,β=π/2)] stands for $|\Phi^{-}\rangle$ ($|\Phi^{+}\rangle$).

Then, the photon 1 (2) is directed to a polarisation rotator of angle $\theta_{1}$ ($\theta_{2}$) and a polarizing beam-splitter (PBS) that transmits (reflects) vertical (horizontal) polarisation. Two photodetectors D1V and D1H (D2V and D2H) are placed at the outgoing channels of PBS1 (PBS2).

The situation $\theta_{1}=\theta_{2}=0$ ($\theta_{1}=\theta_{2}=\pi /4$) corresponds to the measurement in the rectilinear (diagonal) basis. Only in these two cases, a partial analysis of Bell states can be achieved. Concretely, if the rectilinear basis is used, a joint detection in D1H and D2V, or D1V and D2H, would identify the class $\{|\Psi^{+}\rangle ,|\Psi^{-}\rangle \}$. In contrast, a detection in D1H and D2H, or D1V and D2V, would correspond to the class $\{|\Phi^{+}\rangle ,|\Phi^{-}\rangle \}$. Hence, in the rectilinear analysis, it is not possible to determine the value of κ. Nevertheless, if the diagonal basis is used, a detection in D1H and D2V, or D1V and D2H, would identify the class $\{|\Psi^{-}\rangle ,|\Phi^{-}\rangle \}$. In contrast, a detection in D1H and D2H, or D1V and D2V would correspond to the class $\{|\Psi^{+}\rangle ,|\Phi^{+}\rangle \}$. Each of the two analyses gives the maximal distinguishability in this kind of experiment, in which the two photons are not brought together at the apparatus, and corresponds to the possibility of distinguishing two classes of Bell states. On the other hand, if two consecutive experiments are carried out by starting from the same initial state, one of them in the rectilinear basis and the other one in the diagonal one, a complete distinguishability of the four polarisation Bell states is possible.

## 3. WRHP Description of Entanglement Generation Using Type-II PDC

In the WRHP approach, photons are just wave packets generated via the interaction into the crystal between the zeropoint field and a classical wave corresponding to the laser pumping. The Wigner formalism emphasises the undulatory nature of light, so that the information is not carried out by a particle but storaged into the electromagnetic field. Let us now review the basic aspects of the WRHP approach applied to the polarisation entanglement generated via type-II PDC [2]. The crystal is pumped by a laser field, which is represented by a monochromatic plane wave:

(3) $$V_{p}\left(r,t\right)=V_{p}e^{i(k_{0}\cdot r-\omega_{0}t)}+c.c.\;\;,$$

and two zeropoint input beams, which are represented by the stochastic fields:

(4) $$E_{vj}\left(r,t\right)=E_{vj}^{\left(+\right)}\left(r,t\right)+E_{vj}^{\left(-\right)}\left(r,t\right),$$

where

(5) $$\begin{array}{c} E_{vj}^{\left(+\right)}\left(r,t\right)=i\sum_{k\in {\left[k\right]}_{j},\lambda }{\left(\frac{\hbar \omega_{k}}{2\epsilon_{0}L^{3}}\right)}^{\frac{1}{2}}\alpha_{k,\lambda }\epsilon_{k,\lambda }e^{i(k\cdot r-\omega_{k}t)}\;\;\;;\;\;\;j=\left\{1,2\right\}. \end{array}$$

From now on, the lowercase letter “v” will refer to the vacuum or zeropoint field. The sum is restricted to the set of wave vectors centered at a given $k_{j}$, with frequencies $\omega_{k}\approx \omega_{j}$, being $\omega_{k}=c\left|k\right|$, and λ={H,V} represents polarisation. On the other hand, $L^{3}$ is the normalisation volume and $\epsilon_{k,\lambda }$ is a unit polarisation vector. The wave vectors and frequencies corresponding to the zeropoint beams described in Equation (5) fulfill the matching conditions, so that if $k\in {\left[k\right]}_{1}$ and $k^{\prime }\in {\left[k\right]}_{2}$, then $\omega_{k}+\omega_{k^{\prime }}\approx \omega_{0}$ and $k+k^{\prime }\approx k_{0}$. Given that each of the input fields contains two sets of orthogonal vacuum modes, the number of sets of ZPF modes that are amplified at the crystal is equal to four.

The set of zeropoint amplitudes, $\alpha \equiv \{\alpha_{kand\lambda }\}$, are distributed according the Wigner function for the vacuum state, and correspond to the Gaussian:

(6) $$W_{\mathrm{ZPF}}\left(\alpha \right)=\prod_{k,\lambda }W_{\mathrm{ZPF}}\left(\alpha_{k,\lambda }\right)\;\;\;;\;\;\;W_{\mathrm{ZPF}}\left(\alpha_{k,\lambda }\right)=\frac{2}{\pi }e^{-2|\alpha_{k,\lambda }{|}^{2}}.$$

By putting $\alpha_{k,\lambda }=\left|\alpha_{k,\lambda }\right|\exp\left(i\varphi_{k,\lambda }\right)$, the marginal probability distributions of $|\alpha_{k,\lambda }|$ and $\varphi_{k,\lambda }$ can be easily calculated. By considering that $d\alpha_{k,\lambda }=|\alpha_{k,\lambda }\left|d\right|\alpha_{k,\lambda }|d\varphi_{k,\lambda }$, we have:

(7) $$w_{1}\left(\right|\alpha_{k,\lambda }\left|\right)=\int_{0}^{2\pi }W_{\mathrm{ZPF}}\left(\alpha_{k,\lambda }\right)d\varphi_{k,\lambda }=2\pi W_{\mathrm{ZPF}}\left(\alpha_{k,\lambda }\right)=4e^{-2|\alpha_{k,\lambda }{|}^{2}},$$

(8) $$w_{2}\left(\varphi_{k,\lambda }\right)=\int_{0}^{\infty }W_{\mathrm{ZPF}}\left(\alpha_{k,\lambda }\right)|\alpha_{k,\lambda }\left|d\right|\alpha_{k,\lambda }|=\frac{1}{2\pi },$$

where the integral expression

(9) $$\int_{0}^{\infty }x^{2n+1}e^{-bx^{2}}\;dx=\frac{n!}{2b^{n+1}},$$

has been taken into account. In addition, from Equations (6)–(8), it can be observed that $|\alpha_{k,\lambda }|$ and $\varphi_{k,\lambda }$ are independent. That is,

(10) $$W_{\mathrm{ZPF}}\left(\alpha_{k,\lambda }\right)=w_{1}\left(\right|\alpha_{k,\lambda }\left|\right)w_{2}\left(\varphi_{k,\lambda }\right).$$

Given two complex amplitudes, A(r,t;α) and $B(r^{\prime },t^{\prime };\alpha )$, the correlation between them is given by:

(11) $$\langle AB\rangle \equiv \int W_{\mathrm{ZPF}}\left(\alpha \right)A\left(r,t;\alpha \right)B\left(r^{\prime },t^{\prime };\alpha \right)d\alpha .$$

For instance, from (6), the well known correlation properties hold:

(12) $$\langle \alpha_{k,\lambda }\alpha_{k^{\prime },\lambda^{\prime }}\rangle =\langle \alpha_{k,\lambda }^{*}\alpha_{k^{\prime },\lambda^{\prime }}^{*}\rangle =0$$

(13) $$\langle \alpha_{k,\lambda }\alpha_{k^{\prime },\lambda^{\prime }}^{*}\rangle =\frac{1}{2}\delta_{k,k^{\prime }}\delta_{\lambda ,\lambda^{\prime }}.$$

Equations (12) and (13) are closely related to the randomness of the vacuum phases.

The zeropoint beams $E_{v1}^{\left(+\right)}=\left(E_{v1,H}^{\left(+\right)},E_{v1,V}^{\left(+\right)}\right)$ and $E_{v2}^{\left(+\right)}=\left(E_{v2,H}^{\left(+\right)},E_{v2,V}^{\left(+\right)}\right)$ couple with the laser pumping into the nonlinear source giving rise to two correlated beams $E_{1}^{\left(+\right)}$ and $E_{2}^{\left(+\right)}$. On the other hand, the field amplitudes are usually expressed in terms of slowly varying amplitudes (see on the left side of the Figure 1):

(14) $$F_{j}^{\left(+\right)}=e^{i\omega_{j}t}E_{j}^{\left(+\right)}\;\;\;\;;\;\;\;\;j=\left\{1,2\right\}.$$

The exiting fields at the centre of the nonlinear source are expressed by means of the following amplitudes (for simplicity, the space–time notation will be discarded):

(15) $$F_{1}^{\left(+\right)}=\left[\begin{array}{c} F_{1H}^{\left(+\right)}\\ F_{1V}^{\left(+\right)} \end{array}\right]=\left[\begin{array}{c} (1+g^{2}{\left|V\right|}^{2}J)F_{v1,H}^{\left(+\right)}+gVGF_{v2,V}^{\left(-\right)}\\ (1+g^{2}{\left|V\right|}^{2}J)F_{v1,V}^{\left(+\right)}+gVGF_{v2,H}^{\left(-\right)} \end{array}\right],$$

(16) $$F_{2}^{\left(+\right)}=\left[\begin{array}{c} F_{2H}^{\left(+\right)}\\ F_{2V}^{\left(+\right)} \end{array}\right]=\left[\begin{array}{c} (1+g^{2}{\left|V\right|}^{2}J)F_{v2,H}^{\left(+\right)}+gVGF_{v1,V}^{\left(-\right)}\\ \;(1+g^{2}{\left|V\right|}^{2}J)F_{v2,V}^{\left(+\right)}+gVGF_{v1,H}^{\left(-\right)} \end{array}\right],$$

where *g* is a coupling parameter, and *G* and *J* are linear operators. On the other hand, $V=V_{p}/\sqrt{2}$, in order to consider that the energy of the classical wave corresponding to the laser, which is proportional to the squared amplitude, must be divided into four amplitudes.

Equations (15) and (16) represent the WRHP description of two beams whose correlation properties reproduce the theoretical predictions of the state $|\Psi^{+}\rangle$ (see Equation (1)). As a matter of fact, the correlation properties of the vacuum amplitudes, represented by Equations (12) and (13), give rise to a correlation between $F_{1H}^{\left(+\right)}$ and $F_{2V}^{\left(+\right)}$, and an identical correlation between $F_{1V}^{\left(+\right)}$ and $F_{2H}^{\left(+\right)}$, that identify entanglement in the Wigner formalism. Each of these correlations is calculated, to the first order in the coupling constant, and expressed through the sum of two equal addends, each of them involving the zeroth order term of one field and the first order term of the other.

## 4. The Experiment: Field Amplitudes at the Detectors

Let us now consider the joint action of a wave retarder (κ) and a polarisation rotator (β) on beam 1, each of them introducing two possible elements of classical information. By considering the possibilities κ={0,π} and β={0,π/2}, four situations can be generated, each one corresponding to a given Bell state (see Equations (1) and (2)). The joint action of these two apparatuses gives rise to a field ${F^{\prime }}_{1}^{\left(+\right)}$, given by:

(17) $${F^{\prime }}_{1}^{\left(+\right)}=\tilde{P}\left(\beta ,\kappa \right)F_{1}^{\left(+\right)}\;\;\;\;;\;\;\;\;\tilde{P}\left(\beta ,\kappa \right)=\hat{R}\left(\beta \right)\hat{M}\left(\kappa \right),$$

where $\tilde{P}\left(\beta ,\kappa \right)$ is the matrix corresponding to the preparer, $\hat{R}\left(\beta \right)$ represents a rotation of angle β and $\hat{M}\left(\kappa \right)$ represents a wave retarder of phase κ. That is,

(18) $$\begin{array}{c} \tilde{P}\left(\beta ,\kappa \right)=\left[\begin{array}{c} \cos\beta \;\;\;\;\;-e^{i\kappa }\sin\beta \\ \sin\beta \;\;\;\;\;\;\;\;\;\;\;e^{i\kappa }\sin\beta \end{array}\right]. \end{array}$$

Once beam 1 is modified, the polarisation is measured on beam 1 (2) by using a polarisation rotator of angle $\theta_{1}$ ($\theta_{2}$), a polarizing beam-splitter that transmits (reflects) vertical (horizontal) polarisation and two detectors. This corresponds to the use of a different basis for the measurement of the polarisation on beams 1 and 2. The field amplitudes entering PBS1 (see Equation (17)) and PBS2 are:

(19) $${F^{\prime \prime }}_{1}^{\left(+\right)}=\hat{R}\left(\theta_{1}\right){F^{\prime }}_{1}^{\left(+\right)}=\hat{R}\left(\beta +\theta_{1}\right)\hat{M}\left(\kappa \right)F_{1}^{\left(+\right)},$$

(20) $${F^{\prime }}_{2}^{\left(+\right)}=\hat{R}\left(\theta_{2}\right)F_{2}^{\left(+\right)}.$$

By substituting Equations (15) and (16) into (19) and (20), respectively, the field amplitudes entering the PBSs are given by:

(21) $${F^{\prime \prime }}_{1}^{\left(+\right)}=\left[\begin{array}{c} F_{1H}^{\prime \prime (+)}\\ F_{1V}^{\prime \prime (+)} \end{array}\right]=\left[\begin{array}{c} {}[(1+g^{2}{\left|V\right|}^{2}J)F_{v1,H}^{\left(+\right)}+gVGF_{v2,V}^{\left(-\right)}]\cos\left(\theta_{1}+\beta \right)\\ -[(1+g^{2}{\left|V\right|}^{2}J)F_{v1,V}^{\left(+\right)}+gVGF_{v2,H}^{\left(-\right)}]e^{i\kappa }\sin\left(\theta_{1}+\beta \right)\\ \;[(1+g^{2}{\left|V\right|}^{2}J)F_{v1,H}^{\left(+\right)}+gVGF_{v2,V}^{\left(-\right)}]\sin(\theta_{1}+\beta )\\ +[(1+g^{2}{\left|V\right|}^{2}J)F_{v1,V}^{\left(+\right)}+gVGF_{v2,H}^{\left(-\right)}]e^{i\kappa }\cos\left(\theta_{1}+\beta \right) \end{array}\right],$$

(22) $${F^{\prime }}_{2}^{\left(+\right)}=\left[\begin{array}{c} F_{2H}^{\prime (+)}\\ F_{2V}^{\prime (+)} \end{array}\right]=\left[\begin{array}{c} {}[(1+g^{2}{\left|V\right|}^{2}J)F_{v2,H}^{\left(+\right)}+gVGF_{v1,V}^{\left(-\right)}]\cos\theta_{2}\\ -[(1+g^{2}{\left|V\right|}^{2}J)F_{v2,V}^{\left(+\right)}+gVGF_{v1,H}^{\left(-\right)}]\sin\theta_{2}\\ \;[(1+g^{2}{\left|V\right|}^{2}J)F_{v2,H}^{\left(+\right)}+gVGF_{v1,V}^{\left(-\right)}]\sin\theta_{2}\\ +[(1+g^{2}{\left|V\right|}^{2}J)F_{v2,V}^{\left(+\right)}+gVGF_{v1,H}^{\left(-\right)}]\cos\theta_{2} \end{array}\right].$$

In the following, a four-mode approximation will be used, so that *G* and *J* will be replaced by 1 and 1/2, respectively [1]. In addition, in order to investigate the role of the vacuum phases in the Bell-state analysis, Equations (21) and (22) will be modified by putting *V* = |V|exp(iθ) and $F_{vj,X}^{\left(+\right)}$ =$|F_{vj,X}^{\left(+\right)}|\exp\left(i\varphi_{vj,X}\right)$ with j={1,2} and X={H,V}. That is,

(23) $${F{}^{\prime }{}^{\prime }}_{1}^{\left(+\right)}=\left[\begin{array}{c} F_{1H}^{\prime \prime (+)}\\ F_{1V}^{\prime \prime (+)} \end{array}\right]=\left[\begin{array}{c} |F_{v1,H}^{\left(+\right)}|e^{i\varphi_{v1,H}}\cos\left(\theta_{1}+\beta \right)-\left|F_{v1,V}^{\left(+\right)}\right|e^{i(\varphi_{v1,V}+\kappa )}\sin\left(\theta_{1}+\beta \right)\\ +gV\left[|F_{v2,V}^{\left(+\right)}|e^{-i(\varphi_{v2,V}-\theta )}\cos\left(\theta_{1}+\beta \right)-\left|F_{v2,H}^{\left(+\right)}\right|e^{-i(\varphi_{v2,H}-\theta -\kappa )}\sin\left(\theta_{1}+\beta \right)\right]\\ +\frac{g^{2}{\left|V\right|}^{2}}{2}\left[|F_{v1,H}^{\left(+\right)}|e^{i\varphi_{v1,H}}\cos\left(\theta_{1}+\beta \right)-\left|F_{v1,V}^{\left(+\right)}\right|e^{i(\varphi_{v1,V}+\kappa )}\sin\left(\theta_{1}+\beta \right)\right]\\ |F_{v1,H}^{\left(+\right)}|e^{i\varphi_{v1,H}}\sin(\theta_{1}+\beta )+\left|F_{v1,V}^{\left(+\right)}\right|e^{i(\varphi_{v1,V}+\kappa )}\cos\left(\theta_{1}+\beta \right)\\ +gV\left[|F_{v2,V}^{\left(+\right)}|e^{-i(\varphi_{v2,V}-\theta )}\sin(\theta_{1}+\beta )+\left|F_{v2,H}^{\left(+\right)}\right|e^{-i(\varphi_{v2,H}-\theta -\kappa )}\cos\left(\theta_{1}+\beta \right)\right]\\ +\frac{g^{2}{\left|V\right|}^{2}}{2}\left[|F_{v1,H}^{\left(+\right)}|e^{i\varphi_{v1,H}}\sin\left(\theta_{1}+\beta \right)+\left|F_{v1,V}^{\left(+\right)}\right|e^{i(\varphi_{v1,V}+\kappa )}\cos\left(\theta_{1}+\beta \right)\right] \end{array}\right],$$

(24) $${F^{\prime }}_{2}^{\left(+\right)}=\left[\begin{array}{c} F_{2H}^{\prime (+)}\\ F_{2V}^{\prime (+)} \end{array}\right]=\left[\begin{array}{c} |F_{v2,H}^{\left(+\right)}|e^{i\varphi_{v2,H}}\cos\theta_{2}-\left|F_{v2,V}^{\left(+\right)}\right|e^{i\varphi_{v2,V}}\sin\theta_{2}\\ +gV\left[|F_{v1,V}^{\left(+\right)}|e^{-i(\varphi_{v1,V}-\theta )}\cos\theta_{2}-\left|F_{v1,H}^{\left(+\right)}\right|e^{-i(\varphi_{v1,H}-\theta )}\sin\theta_{2}\right]\\ +\frac{g^{2}{\left|V\right|}^{2}}{2}\left[|F_{v2,H}^{\left(+\right)}|e^{i\varphi_{v2,H}}\cos\theta_{2}-\left|F_{v2,V}^{\left(+\right)}\right|e^{i\varphi_{v2,V}}\sin\theta_{2}\right]\\ |F_{v2,H}^{\left(+\right)}|e^{i\varphi_{v2,H}}\sin\theta_{2}+\left|F_{v2,V}^{\left(+\right)}\right|e^{i\varphi_{v2,V}}\cos\theta_{2}\\ +gV\left[|F_{v1,V}^{\left(+\right)}|e^{-i(\varphi_{v1,V}-\theta )}\sin\theta_{2}+\left|F_{v1,H}^{\left(+\right)}\right|e^{-i(\varphi_{v1,H}-\theta )}\cos\theta_{2}\right]\\ +\frac{g^{2}{\left|V\right|}^{2}}{2}\left[|F_{v2,H}^{\left(+\right)}|e^{i\varphi_{v2,H}}\sin\theta_{2}+\left|F_{v2,V}^{\left(+\right)}\right|e^{i\varphi_{v2,V}}\cos\theta_{2}\right] \end{array}\right].$$

Presently, by taking into account the action of PBSj on beam *j*, which introduces a vacuum field $F_{ZPFj}^{\left(+\right)}=\left(F_{ZPFj,H}^{\left(+\right)},F_{ZPFj,V}^{\left(+\right)}\right)$, the field amplitudes at the detectors are:

(25) $$F_{D1H}^{\left(+\right)}=\left[\begin{array}{c} iF_{1H}^{\prime \prime (+)}\\ F_{ZPF1,V}^{\left(+\right)} \end{array}\right]\;\;\;;\;\;\;F_{D1V}^{\left(+\right)}=\left[\begin{array}{c} iF_{ZPF1,H}^{\left(+\right)}\\ F_{1V}^{\prime \prime (+)} \end{array}\right]\;\;\;;\;\;\;F_{D2H}^{\left(+\right)}=\left[\begin{array}{c} iF_{2H}^{\prime (+)}\\ F_{ZPF2,V}^{\left(+\right)} \end{array}\right]\;\;\;;\;\;\;F_{D2V}^{\left(+\right)}=\left[\begin{array}{c} iF_{ZPF2,H}^{\left(+\right)}\\ F_{2V}^{\prime (+)} \end{array}\right],$$

where $F_{1H}^{\prime \prime (+)}$ and $F_{1V}^{\prime \prime (+)}$ ($F_{2H}^{\prime (+)}$ and $F_{2V}^{\prime (+)}$) are given in Equations (23) and (24), respectively.

## 5. ZPF Inputs and Distinguishability

As it has been demonstrated elsewhere [4], for two photons entangled in *n* dichotomic degrees of freedom, the number of sets of ZPF modes that are amplified at the source, $N_{ZPF,S}$=$2^{n+1}$, just coincides with the upper bound to maximal distinguishability of Bell states. In the setup described in Figure 1, given that n=1, we have:

(26) $$N_{ZPF,S}=4.$$

In experiments in which there are no additional zeropoint entries between the source and the Bell-state analyser, $N_{ZPF,S}=N_{ZPF,A}$, where $N_{ZPF,A}$ is the number of sets of ZPF modes entering the analyser.

Furthermore, if the two photons are not overlapped at a balanced beam-splitter, the maximal distinguishability in this kind of experiments, $2^{n}$, can be calculated via the difference between the number of sets of zeropoint amplitudes that are amplified at the source and enter the analyser, and the number of sets of zeropoint modes that enter the area of a single detection event. In this case (see Figure 1),

(27) $$N_{sing.det.area}^{noise}=\frac{N_{ZPF,A}^{noise}}{2}=2^{n}=2,$$

$N_{ZPF,A}^{noise}=2^{n+1}=4$ being the total number of sets of ZPF modes entering the idle channels of the PBSs inside the analyser. By taking into consideration Equations (26) and (27), the following result has been obtained elsewhere [1]:

(28) $$N_{max,class}=N_{ZPF,S}-N_{sing.det.area}^{noise}=2^{n}=2.$$

In the experimental situation described in Figure 1, the use of the rectilinear basis ($\theta_{1}=\theta_{2}=0$) allows for the discrimination of the classes $\{|\Psi^{+}\rangle ,|\Psi^{-}\rangle \}$ and $\{|\Phi^{+}\rangle ,|\Phi^{-}\rangle \}$. On the other hand, the classes $\{|\Psi^{+}\rangle ,|\Phi^{+}\rangle \}$ and $\{|\Psi^{-}\rangle ,|\Phi^{-}\rangle \}$ can be distinguished by using a diagonal analysis ($\theta_{1}=\theta_{2}=\pi /4$). In both cases, the number of distinguishable classes corresponds to the difference between the number of sets of ZPF modes that are amplified at the source and the number of sets of ZPF modes entering the single detection area (see Equation (28)).

If two experiments are made consecutively by starting from the same initial state, one of them in the rectilinear basis, and the other in the diagonal one, then a complete distinguishability is possible, i.e., the two dichotomic parameters, β and κ, are recognised through these two experiments. By taking into account that both the number of sets of ZPF modes entering the source and the number of sets of ZPF modes entering the single detection area must be multiplied by a factor 2 in the consideration of the two consecutive experiments in the information-to-noise balance, complete distinguishability is explained from a sufficient balance that allows for the BSM of the four Bell states:

(29) $$N_{max,class}=2\left(N_{ZPS,S}-N_{sing.det.area}^{noise}\right)=8-4=4.$$

## 6. The Signal Intensities at the Detectors

The intensity of light at a given detector DjX is proportional to the squared electric field amplitude, that is $I_{DjX}\left(\alpha ;\phi_{j}\right)\propto F_{DjX}^{\left(+\right)}\cdot F_{DjX}^{\left(-\right)}$, and it depends on the vacuum amplitudes (α) and controllable parameters of the experimental setup corresponding to the propagation of the electric field from the source to the detector area *j*, which are represented by $\phi_{j}$. Given that the electric field amplitudes at the detectors are expressed as linear transformations, to the second order in the coupling constant, of the zeropoint amplitudes entering the source, the intensity can be decomposed as a sum of two terms:

(30) $$I_{DjX}={\tilde{I}}_{DjX}+I_{DjX,ZPF},$$

where $I_{DjX,ZPF}$ is the contribution to the intensity of the pure zeropoint field, i.e., the intensity if the radiation sources were turned off. In the case of PDC, $I_{DjX,ZPF}=I_{DjX}\left(g=0\right)$. On the other hand, ${\tilde{I}}_{DjX}$ is the *signal intensity*, i.e., the part of the total intensity in which the zeropoint contribution has been removed.

The single detection probability in PDC experiments is usually calculated by means of the expression [1]:

(31) $$P_{DjX}\propto \langle I_{DjX}-I_{v,DjX}\rangle ,$$

where $I_{v,DjX}$ is the mean value of the zeropoint intensity at the position of the detector, i.e., $I_{v,DjX}=\langle I_{DjX,ZPF}\rangle$. Therefore, the use of $I_{v,DjX}$ or $I_{DjX,ZPF}$ in Equation (31) gives rise to the same result for the single detection probability, resulting in:

(32) $$P_{DjX}\propto \langle {\tilde{I}}_{DjX}\left(\alpha ;\phi_{j}\right)\rangle .$$

Let us now consider the joint detection probability corresponding to two detectors, D1X and $D2X^{\prime }$. If the electric field operators corresponding to positions $r_{D1X}$ and $r_{D2X^{\prime }}$ commute, as in the case of Bell-type experiments, the joint detection probability is given by the expression:

(33) $$P_{D1X,D2X^{\prime }}\propto \langle \left[I_{D1X}\left(\alpha ;\phi_{1}\right)-I_{v,D1X}\right]\left[I_{D2X^{\prime }}\left(\alpha ;\phi_{2}\right)-I_{v,D2X^{\prime }}\right]\rangle ,$$

where the mean value of the zeropoint intensity at each detector must be removed. At this point, it must be stressed that $I_{v,DjX}$ cannot be substituted by $I_{DjX,ZPF}$ in Equation (33), so that $\langle \left[I_{D1X}-I_{v,D1X}\right]\left[I_{D2X^{\prime }}-I_{v,D2X^{\prime }}\right]\rangle \neq \langle {\tilde{I}}_{D1X}{\tilde{I}}_{DjX^{\prime }}\rangle$.

In PDC experiments involving polarisation, the following expression is usually used for calculation purposes, in which the joint detection probability is calculated in terms of the field amplitudes [2]:

(34) $$P_{D1X,D2X^{\prime }}\propto \sum_{\lambda }\sum_{\lambda^{\prime }}{\left|F_{D1X,\lambda }^{\left(+\right)}\left(\alpha ;\phi_{1}\right)F_{D2X^{\prime },\lambda^{\prime }}^{\left(+\right)}\left(\alpha ;\phi_{2}\right)\right|}^{2}.$$

The use of the former expression in the calculation of the joint detection probabilities, in the experimental setup given in Figure 1, gives rise to the known results described in Section 2. As a matter of fact, a similar experiment has been studied elsewhere with the WRHP formalism, concerning the polarisation encoding the quantum key distribution [3].

In this paper, no calculation of the joint detection rates will be made. In contrast, the main results of this work will emerge from granting a main role to the signal intensities at the detectors as functions of the modulus and phases of the vacuum amplitudes.

By using Equations (23)–(25) and (30), the signal intensities and the zeropoint intensities have been calculated as functions of the modules and phases of the zeropoint amplitudes and the experimental parameters. The dependence with the four vacuum phases $\varphi_{vj,X}$ (j={1,2} and X={H,V}) is represented by the six phase combinations $\varphi_{v1,H}+\varphi_{v2,V}-\theta$, $\varphi_{v1,V}+\varphi_{v2,H}-\theta$, $\varphi_{v1,H}+\varphi_{v2,H}-\theta$, $\varphi_{v1,V}+\varphi_{v2,V}-\theta$, $\varphi_{v2,H}-\varphi_{v2,V}$ and $\varphi_{v1,H}-\varphi_{v1,V}$, from which only three of them are independent. For instance, by defining the following phases:

(35) $$\Omega_{1}\equiv \varphi_{v1,H}+\varphi_{v2,V}-\theta ,$$

(36) $$\Omega_{2}\equiv \varphi_{v1,V}+\varphi_{v2,H}-\theta ,$$

(37) $$\Omega_{3}\equiv \varphi_{v1,H}+\varphi_{v2,H}-\theta ,$$

the rest of the combinations can be expressed as:

(38) $$\varphi_{v1,V}+\varphi_{v2,V}-\theta =\Omega_{1}+\Omega_{2}-\Omega_{3},$$

(39) $$\varphi_{v2,H}-\varphi_{v2,V}=\Omega_{3}-\Omega_{1},$$

(40) $$\varphi_{v1,H}-\varphi_{v1,V}=\Omega_{3}-\Omega_{2}.$$

Phases $\Omega_{1}$ and $\Omega_{2}$ have been defined elsewhere (see Equation (50) of [1]), and they arose from the analysis of the experiment in the rectilinear basis. The appearance of new phases is a consequence of the consideration of a generic experimental context, in which $\theta_{1}$ and $\theta_{2}$ can take any value.

The zeropoint intensities are:

(41) $$\begin{array}{c} I_{D1H,ZPF}=|F_{ZPF1,V}^{\left(+\right)}{|}^{2}+\cos^{2}\left(\beta +\theta_{1}\right)|F_{v1,H}^{\left(+\right)}{|}^{2}+\sin^{2}\left(\beta +\theta_{1}\right){\left|F_{v1,V}^{\left(+\right)}\right|}^{2}\\ -\sin\left[2\left(\beta +\theta_{1}\right)\right]\cos\kappa |F_{v1,H}^{\left(+\right)}\left|\right|F_{v1,V}^{\left(+\right)}|\cos\left(\Omega_{3}-\Omega_{2}\right), \end{array}$$

(42) $$\begin{array}{c} I_{D1V,ZPF}=|F_{ZPF1,H}^{\left(+\right)}{|}^{2}+\sin^{2}\left(\beta +\theta_{1}\right)|F_{v1,H}^{\left(+\right)}{|}^{2}+\cos^{2}\left(\beta +\theta_{1}\right){\left|F_{v1,V}^{\left(+\right)}\right|}^{2}\\ +\sin\left[2\left(\beta +\theta_{1}\right)\right]\cos\kappa |F_{v1,H}^{\left(+\right)}\left|\right|F_{v1,V}^{\left(+\right)}|\cos\left(\Omega_{3}-\Omega_{2}\right), \end{array}$$

(43) $$\begin{array}{c} I_{D2H,ZPF}=|F_{ZPF2,V}^{\left(+\right)}{|}^{2}+\cos^{2}\theta_{2}|F_{v2,H}^{\left(+\right)}{|}^{2}+\sin^{2}\theta_{2}{\left|F_{v2,V}^{\left(+\right)}\right|}^{2}\\ -\sin\left(2\theta_{2}\right)|F_{v2,H}^{\left(+\right)}\left|\right|F_{v2,V}^{\left(+\right)}|\cos\left(\Omega_{3}-\Omega_{1}\right), \end{array}$$

(44) $$\begin{array}{c} I_{D2V,ZPF}=|F_{ZPF2,H}^{\left(+\right)}{|}^{2}+\sin^{2}\theta_{2}|F_{v2,H}^{\left(+\right)}{|}^{2}+\cos^{2}\theta_{2}{\left|F_{v2,V}^{\left(+\right)}\right|}^{2}\\ +\sin\left(2\theta_{2}\right)|F_{v2,H}^{\left(+\right)}\left|\right|F_{v2,V}^{\left(+\right)}|\cos\left(\Omega_{3}-\Omega_{1}\right), \end{array}$$

where each zeropoint contribution to the total intensity, $I_{DjX,ZPF}$, contains a term related to the zeropoint input at the vacuum channel of the corresponding PBS, $|F_{ZPFj,X_{⊥}}^{\left(+\right)}{|}^{2}$, $X_{⊥}$ being the orthogonal polarisation to *X*.

In order to express the signal intensities in a simplified form, the following functions of the zeropoint amplitudes and the crystal and laser parameters will be defined:

(45) $$R_{1}\left(\right|F_{v1,H}^{\left(+\right)}\left|,\right|F_{v2,V}^{\left(+\right)}|;\Omega_{1})\equiv 2g|V||F_{v1,H}^{\left(+\right)}\left|\right|F_{v2,V}^{\left(+\right)}|\cos\Omega_{1}+g^{2}{\left|V\right|}^{2}\left(\right|F_{v1,H}^{\left(+\right)}{|}^{2}+|F_{v2,V}^{\left(+\right)}{|}^{2}),$$

(46) $$R_{2}\left(\right|F_{v1,V}^{\left(+\right)}\left|,\right|F_{v2,H}^{\left(+\right)}|;\Omega_{2})\equiv 2g|V||F_{v1,V}^{\left(+\right)}\left|\right|F_{v2,H}^{\left(+\right)}|\cos\Omega_{2}+g^{2}{\left|V\right|}^{2}\left(\right|F_{v1,V}^{\left(+\right)}{|}^{2}+|F_{v2,H}^{\left(+\right)}{|}^{2}),$$

(47) $$\begin{array}{c} D(|F_{v1,H}^{\left(+\right)}|,|F_{v1,V}^{\left(+\right)}|,|F_{v2,H}^{\left(+\right)}|,|F_{v2,V}^{\left(+\right)}|;\Omega_{1},\Omega_{2},\Omega_{3})\\ \equiv g|V|\left[|F_{v1,H}^{\left(+\right)}\left|\right|F_{v2,H}^{\left(+\right)}|\cos\Omega_{3}+|F_{v1,V}^{\left(+\right)}\left|\right|F_{v2,V}^{\left(+\right)}|\cos\left(\Omega_{1}+\Omega_{2}-\Omega_{3}\right)\right]\\ +g^{2}{\left|V\right|}^{2}\left[|F_{v1,H}^{\left(+\right)}\left|\right|F_{v1,V}^{\left(+\right)}|\cos\left(\Omega_{3}-\Omega_{2}\right)+|F_{v2,H}^{\left(+\right)}\left|\right|F_{v2,V}^{\left(+\right)}|\cos\left(\Omega_{3}-\Omega_{1}\right)\right]. \end{array}$$

In terms of the functions $R_{1}$, $R_{2}$ and *D*, and the preparation (β, κ) and measurement ($\theta_{1}$, $\theta_{2}$) parameters, the signal intensities at the detectors have been calculated, resulting in:

(48) $${\tilde{I}}_{D1H}=R_{1}\cos^{2}\left(\beta +\theta_{1}\right)+R_{2}\sin^{2}\left(\beta +\theta_{1}\right)-D\sin\left[2\left(\theta_{1}+\beta \right)\right]\cos\kappa ,$$

(49) $${\tilde{I}}_{D1V}=R_{1}\sin^{2}\left(\beta +\theta_{1}\right)+R_{2}\cos^{2}\left(\beta +\theta_{1}\right)+D\sin\left[2\left(\theta_{1}+\beta \right)\right]\cos\kappa ,$$

(50) $${\tilde{I}}_{D2H}=R_{1}\sin^{2}\theta_{2}+R_{2}\cos^{2}\theta_{2}-D\sin\left(2\theta_{2}\right),$$

(51) $${\tilde{I}}_{D2V}=R_{1}\cos^{2}\theta_{2}+R_{2}\sin^{2}\theta_{2}+D\sin\left(2\theta_{2}\right).$$

At this point, the following properties concerning Equations (41) to (51) should be emphasised for further consideration:

- The factor ${\left(-1\right)}^{n(X)}\cos\kappa \sin\left[2\left(\beta +\theta_{1}\right)\right]$, where n(X)=1 if X=H and n(X)=0 if X=V, takes opposite values in the zeropoint intensities $I_{D1H,ZPF}$ and $I_{D1V,ZPF}$ (see Equations (41) and (42)), and also in the signal intensities ${\tilde{I}}_{D1H}$ and ${\tilde{I}}_{D1V}$ (see Equations (48) and (49)). Analogously, the factor ${\left(-1\right)}^{n(X)}\sin\left(2\theta_{2}\right)$ takes opposite values in $I_{D2H,ZPF}$ and $I_{D2V,ZPF}$ (see Equations (43) and (44)), and also in ${\tilde{I}}_{D2H}$ and ${\tilde{I}}_{D2V}$ (see Equations (50) and (51)).
- The phases $\Omega_{1}$, $\Omega_{2}$ and $\Omega_{3}$, and the phase combination $\Omega_{1}+\Omega_{2}-\Omega_{3}$, appear in the first order term of ${\tilde{I}}_{DjX}$ (see Equations (45)–(47)). Specifically, the opposite sign first-order terms of ${\tilde{I}}_{DjX}$ and ${\tilde{I}}_{DjX_{⊥}}$ contain the phases $\Omega_{3}$ and $\Omega_{1}+\Omega_{2}-\Omega_{3}$.
- The phase combination $\Omega_{3}-\Omega_{2}$ appears in the opposite sign terms of $I_{D1H,ZPF}$ and $I_{D1V,ZPF}$ (see Equations (41) and (42)), and also in the opposite second order contributions of ${\tilde{I}}_{D1H}$ and ${\tilde{I}}_{D1V}$ (see Equations (47)–(49)).
- Analogously, $\Omega_{3}-\Omega_{1}$ appears in the opposite sign terms of $I_{D2H,ZPF}$ and $I_{D2V,ZPF}$ (see Equations (43) and (44)), and also in the opposite second order contributions of ${\tilde{I}}_{D2H}$ and ${\tilde{I}}_{D2V}$ (see Equations (47), (50) and (51)).

The single detection probabilities at the detectors can be calculated via Equation (32). By averaging the signal intensities given in Equations (48)–(51) and taking into consideration the randomness of the vacuum phases, the contribution of the terms including $\Omega_{i}$ (i=1,2,3) in Equations (45)–(47) is equal to zero. In addition, by considering that $\langle |F_{vj,X}^{\left(+\right)}{|}^{2}\rangle$ takes the same value, independently of *i* and *X*, the mean values of $R_{1}$, $R_{2}$ and *D* are:

(52) $$\langle R_{1}\rangle =\langle R_{2}\rangle =2g^{2}{\left|V\right|}^{2}A\;\;\;\;;\;\;\;\;\langle D\rangle =0,$$

where

(53) $$A\equiv \langle |F_{vj,X}^{\left(+\right)}{|}^{2}\rangle \;\;\;;\;\;\;\forall \;\;j=\left\{1,2\right\},\;\;X=\left\{H,V\right\}.$$

This gives the following result, which is independent of β, κ, $\theta_{1}$ and $\theta_{2}$:

(54) $$P_{DjX}\propto \langle R_{j}\rangle =2g^{2}{\left|V\right|}^{2}A\;\;\;\;;\;\;\;\;j=\left\{1,2\right\},\;\;X=\left\{H,V\right\}.$$

Phases $\Omega_{1}$ and $\Omega_{2}$ play an essential role in the description of entanglement in the WRHP formalism [1]. By adding Equations (48)–(51) the opposite factors are cancelled with each other, and the total signal intensity corresponding to the beam $F_{j}^{\left(+\right)}$ emitted by the crystal (see Equations (15) and (16)) and carried out throughout the setup is:

(55) $$\begin{array}{c} {\tilde{I}}_{beam}={\tilde{I}}_{D1H}+{\tilde{I}}_{D1V}={\tilde{I}}_{D2H}+{\tilde{I}}_{D2V}=R_{1}+R_{2}\\ =2g|V|\left[|F_{v1,H}^{\left(+\right)}\left|\right|F_{v2,V}^{\left(+\right)}|\cos\Omega_{1}+|F_{v1,V}^{\left(+\right)}\left|\right|F_{v2,H}^{\left(+\right)}|\cos\Omega_{2}\right]\\ +g^{2}{\left|V\right|}^{2}\left[|F_{v1,H}^{\left(+\right)}{|}^{2}+|F_{v2,V}^{\left(+\right)}{|}^{2}+|F_{v1,V}^{\left(+\right)}{|}^{2}+{\left|F_{v2,H}^{\left(+\right)}\right|}^{2}\right]. \end{array}$$

The former Equation reveals that the signal intensity corresponding to the beam $F_{j}^{\left(+\right)}$ has a dependence with the vacuum phases through $\Omega_{1}$ and $\Omega_{2}$ (see Equations (35) and (36)) in the first order term, and there is no dependence with the vacuum phases to the second order. Specifically, $\Omega_{1}$ represents the interference between $F_{v1,H}^{\left(+\right)}$ and $gVGF_{v2,V}^{\left(-\right)}$ in the signal intensity corresponding to $F_{1H}^{\left(+\right)}$, and also the interference between $F_{v2,V}^{\left(+\right)}$ and $gVGF_{v1,H}^{\left(-\right)}$ in the signal intensity corresponding to $F_{2V}^{\left(+\right)}$. In the same way, the interference between $F_{v1,V}^{\left(+\right)}$ and $gVGF_{v2,H}^{\left(-\right)}$ ($F_{v2,H}^{\left(+\right)}$ and $gVGF_{v1,V}^{\left(-\right)}$) in the signal intensity corresponding to $F_{1V}^{\left(+\right)}$ ($F_{2H}^{\left(+\right)}$) is represented by $\Omega_{2}$.

If a detector is placed at the path-way of the beam $F_{j}^{\left(+\right)}$, the single detection probability is

(56) $$P_{j}\propto \langle {\tilde{I}}_{DjH}+{\tilde{I}}_{DjV}\rangle =4Ag^{2}{\left|V\right|}^{2}\;\;\;\;;\;\;\;\;j=\left\{1,2\right\}.$$

## 7. Signal Intensities and Distinguishability

In this section, the relationship between the distinguishability of Bell states and the signal intensities at the detectors will be investigated. To this purpose, two situations corresponding to the maximal distinguishability are considered: the rectilinear and diagonal analyses. As will be shown below, the signal intensities corresponding to correlated detectors are equal.

### 7.1. Analysis in the Rectilinear Basis

Let us consider the situation $\theta_{1}=\theta_{2}=0$. In this case, $\sin\left[2\left(\beta +\theta_{1}\right)\right]=\sin\left(2\theta_{2}\right)=0$ and ∀β={0,π/2}, so that the information concerning the parameter κ is erased from Equations (48) and (49). Given that the function *D* (see Equation (47)) does not contribute in the rectilinear analysis, the phase information in the signal intensities is reduced to $\Omega_{1}$ and $\Omega_{2}$. We have:

(57) $${\tilde{I}}_{D1H}\left(\beta ,\kappa ;\theta_{1}=0\right)=R_{1}\cos^{2}\beta +R_{2}\sin^{2}\beta ,$$

(58) $${\tilde{I}}_{D1V}\left(\beta ,\kappa ;\theta_{1}=0\right)=R_{1}\sin^{2}\beta +R_{2}\cos^{2}\beta ,$$

(59) $${\tilde{I}}_{D2H}\left(\theta_{2}=0\right)=R_{2},$$

(60) $${\tilde{I}}_{D2V}\left(\theta_{2}=0\right)=R_{1},$$

From Equations (46) and (59), for a given value of $|F_{v2,H}^{\left(+\right)}|$ and $|F_{v1,V}^{\left(+\right)}|$, ${\tilde{I}}_{D2H}$ is an oscillating function of $\Omega_{2}$. In the same way, from Equations (45) and (60), ${\tilde{I}}_{D2V}$ is an oscillating function of $\Omega_{1}$ for a given value of $|F_{v2,V}^{\left(+\right)}|$ and $|F_{v1,H}^{\left(+\right)}|$. Presently, the following two situations are analysed:

- In the case β=0, which corresponds to the states $|\Psi^{\pm }\rangle$, detectors corresponding to the orthogonal polarisations are correlated. In this case, it can be easily observed that
  (61) $${\tilde{I}}_{D1V}\left(\beta =0,\kappa ;\theta_{1}=0\right)={\tilde{I}}_{D2H}\left(\theta_{2}=0\right)=R_{2},$$
  and
  (62) $${\tilde{I}}_{D1H}\left(\beta =0,\kappa ;\theta_{1}=0\right)={\tilde{I}}_{D2V}\left(\theta_{2}=0\right)=R_{1}.$$
- On the other hand, in the case β=π/2, which corresponds to the states $|\Phi^{\pm }\rangle$, detectors corresponding to the same polarisation component are correlated:
  (63) $${\tilde{I}}_{D1V}\left(\beta =\frac{\pi }{2},\kappa ;\theta_{1}=0\right)={\tilde{I}}_{D2V}\left(\theta_{2}=0\right)=R_{1},$$
  (64) $${\tilde{I}}_{D1H}\left(\beta =\frac{\pi }{2},\kappa ;\theta_{1}=0\right)={\tilde{I}}_{D2H}\left(\theta_{2}=0\right)=R_{2}.$$

### 7.2. Analysis in the Diagonal Basis

By substituting the values $\theta_{1}=\theta_{2}=\pi /4$ in Equations (48)–(51), the corresponding signal intensities at the detectors are given by the following expressions:

(65) $${\tilde{I}}_{D1X}\left(\beta ,\kappa ;\theta_{1}=\frac{\pi }{4}\right)=\frac{R_{1}+R_{2}}{2}+{\left(-1\right)}^{n(X)}D\cos\left(2\beta \right)\cos\kappa ,$$

(66) $${\tilde{I}}_{D2X}\left(\theta_{2}=\frac{\pi }{4}\right)=\frac{R_{1}+R_{2}}{2}+{\left(-1\right)}^{n(X)}D,$$

where X={H,V}, being n(H)=1 and n(V)=2. In this case, the analysis of which detectors are correlated must be conducted by studying the parameter cos(2β)cosκ:

- *Case I*. cos(2β)cosκ=1. This situation happens in the case (β=0,κ=0), i.e., the state $|\Psi^{+}\rangle$, and (β=π/2,κ=π) corresponding to $|\Phi^{+}\rangle$. From Equations (65) and (66), it can be easily observed that the correlated detectors are those corresponding to the same polarisation component. That is
  (67) $$\begin{array}{c} {\tilde{I}}_{D1X}\left(\beta =0,\kappa =0;\theta_{1}=\frac{\pi }{4}\right)={\tilde{I}}_{D1X}\left(\beta =\frac{\pi }{2},\kappa =\pi ;\theta_{1}=\frac{\pi }{4}\right)\\ ={\tilde{I}}_{D2X}\left(\theta_{2}=\frac{\pi }{4}\right)=\frac{R_{1}+R_{2}}{2}+{\left(-1\right)}^{n(X)}D. \end{array}$$
- *Case II*. cos(2β)cosκ=−1. The two possibilities are (β=0,κ=π), i.e., the state $|\Psi^{-}\rangle$, and (β=π/2,κ=0), which corresponds to the state $|\Phi^{-}\rangle$. From Equations (65) and (66), the joint detection corresponds to detectors having orthogonal polarisation components. We have,
  (68) $$\begin{array}{c} {\tilde{I}}_{D1X}\left(\beta =0,\kappa =\pi ;\theta_{1}=\frac{\pi }{4}\right)={\tilde{I}}_{D1X}\left(\beta =\frac{\pi }{2},\kappa =0;\theta_{1}=\frac{\pi }{4}\right)\\ ={\tilde{I}}_{D2X_{⊥}}\left(\theta_{2}=\frac{\pi }{4}\right)=\frac{R_{1}+R_{2}}{2}-{\left(-1\right)}^{n(X_{⊥})}D, \end{array}$$
  $X_{⊥}$ being the orthogonal polarisation to *X*.

The former analyses give rise to one of the main results of this paper:

*“The signal intensities corresponding to correlated detectors, as functions of the crystal and laser parameters, and the modules and phases of the zeropoint amplitudes that intervene in the experiment, are equal”.*

This result sharply contrasts with the standard approach based on the calculation of the joint detection rates in order to establish which detectors are correlated.

## 8. Extracting Phase Information from the Zeropoint Field

The subtraction of the two sets of ZPF modes entering a single detection area, from the four sets of ZPF modes entering the source (see Equation (28)), gives the maximal distinguishability of the polarisation of Bell states in the experimental setup described in Figure 1. This reflects the relationship between the measurement (i.e., detection) and the subtraction of the zeropoint background (see Equations (31) and (32)).

More concretely, the zeropoint intensity at the detector DjX, $I_{DjX,ZPF}$ (j={1,2}, X={H,V}) (see Equations (41)–(44)), contains an addend related to the zeropoint input at the idle channel of the corresponding PBS, $|F_{ZPFj,X_{⊥}}^{\left(+\right)}{|}^{2}$, where $X_{⊥}$ is the orthogonal polarisation to *X*. These stochastic contributions to the zeropoint intensities are different from each other, because they involve uncorrelated modes. As a consequence, even when the signal intensities at two correlated detectors are equal, the total intensities are not. By taking into consideration that the zeropoint amplitudes that are amplified at the crystal carry the quantum information through the setup, from the source to the detectors, the zeropoint entries at the detection areas act as sources of noise. In other words, the information concerning the zeropoint amplitudes entering the crystal and the preparation parameters remains hidden before detection.

Given that the access to the values of β and κ is only through detection, and detection implies the subtraction of the zeropoint intensity, the maximal distinguishability is related to the subtraction of a sufficient zeropoint contribution coming from the measuring apparatus, from the number of sets of ZPF modes that are amplified at the source, in which the quantum information is stored. Why one half of the total noise represented by the four sets of ZPF modes? Because once the first detection event has been produced, by removing the noise contribution of the other single detection area and observing the signal intensities, one could distinguish which signal intensity fits with the first detection, and so to establish which detectors are correlated. Thus, the idle channels of the PBSs have an effect of limiting the capacity for extracting information.

The question arises of whether the total information extracted via a Bell state analysis could fit to some piece of information drawn from the zeropoint field entering the source. Even though the vacuum amplitudes are intrinsically stochastic, so that the predictions concerning single and joint detection rates in the WRHP formalism (see Equations (31)–(34)) involve the calculation of averages by using the Wigner function of the zeropoint field given in Equation (6), from now on a different approach will be considered.

The signal intensities at the detectors have been calculated in the cases of rectilinear and diagonal basis, as functions of the modules and phases of the zeropoint field amplitudes. Given that entanglement is a property directly related to phase coupling, from now on Equations (57)–(60), (65) and (66), will be substituted by their corresponding averages, by integrating with respect to the modules of the vacuum amplitudes. From now on, the abbreviation SIAM (signal intensity averaged in the modules) will be used.

Therefore, Equations (45)–(47) must be conveniently modified. It follows that:

(69) $${\langle R_{1}\rangle }_{\left|\right|}\left(\Omega_{1}\right)=2g|V|B^{2}\cos\Omega_{1}+2g^{2}{\left|V\right|}^{2}A^{\prime },$$

(70) $${\langle R_{2}\rangle }_{\left|\right|}\left(\Omega_{2}\right)=2g|V|B^{2}\cos\Omega_{2}+2g^{2}{\left|V\right|}^{2}A^{\prime },$$

(71) $$\begin{array}{c} {\langle D\rangle }_{\left|\right|}\left(\Omega_{1},\Omega_{2},\Omega_{3}\right)=g\left|V\right|B^{2}\left\{\cos\Omega_{3}+\cos\left(\Omega_{1}+\Omega_{2}-\Omega_{3}\right)\right.\\ \left.+g|V|[\cos\left(\Omega_{3}-\Omega_{2}\right)+\cos\left(\Omega_{3}-\Omega_{1}\right)]\right\}, \end{array}$$

where the following parameters have been defined:

(72) $$B\equiv \langle |F_{vj,X}^{\left(+\right)}{|\rangle }_{\left|\right|}\;\;\;;\;\;\;A^{\prime }\equiv \langle |F_{vj,X}^{\left(+\right)}{{|}^{2}\rangle }_{\left|\right|}\;\;\;;\;\;\;j=\left\{1,2\right\},\;\;X=\left\{H,V\right\}.$$

${\langle \ldots \rangle }_{\left|\right|}$ denotes averaging over the modules of the vacuum amplitudes. By using Equation (10), it can be easily observed that $\langle |F_{vj,X}^{\left(+\right)}{|}^{2}\rangle_{\left|\right|}=\langle |F_{vj,X}^{\left(+\right)}{|}^{2}\rangle /\left(2\pi \right)$, so that $A^{\prime }=A/\left(2\pi \right)$, where *A* is given in Equation (53). On the other hand, by using Equations (5), (6) and (13), and the following integral expression for the calculation of *B*:

(73) $$\int_{0}^{\infty }x^{2n}e^{-bx^{2}}\;dx=\frac{(2n-1)!!}{b^{n}2^{n+1}}\sqrt{\frac{\pi }{b}},$$

the following relationship between $B^{2}$ and $A^{\prime }$ is obtained:

(74) $$\frac{B^{2}}{A^{\prime }}=\frac{1}{8}.$$

In terms of Equations (69)–(71), the expressions for the SIAMs in the rectilinear basis (see Equations (57)–(60)), as functions of the vacuum phases and the crystal and laser parameters, are:

(75) $${\langle {\tilde{I}}_{D1H}\rangle }_{\left|\right|}\left(\beta ,\kappa ;\theta_{1}=0;\Omega_{1},\Omega_{2}\right)={\langle R_{1}\rangle }_{\left|\right|}\left(\Omega_{1}\right)\cos^{2}\beta +{\langle R_{2}\rangle }_{\left|\right|}\left(\Omega_{2}\right)\sin^{2}\beta ,$$

(76) $${\langle {\tilde{I}}_{D1V}\rangle }_{\left|\right|}\left(\beta ,\kappa ;\theta_{1}=0;\Omega_{1},\Omega_{2}\right)={\langle R_{1}\rangle }_{\left|\right|}\left(\Omega_{1}\right)\sin^{2}\beta +{\langle R_{2}\rangle }_{\left|\right|}\left(\Omega_{2}\right)\cos^{2}\beta ,$$

(77) $${\langle {\tilde{I}}_{D2H}\rangle }_{\left|\right|}\left(\theta_{2}=0;\Omega_{2}\right)={\langle R_{2}\rangle }_{\left|\right|}\left(\Omega_{2}\right),$$

(78) $${\langle {\tilde{I}}_{D2V}\rangle }_{\left|\right|}\left(\theta_{2}=0;\Omega_{1}\right)={\langle R_{1}\rangle }_{\left|\right|}\left(\Omega_{1}\right),$$

and the corresponding ones in the diagonal basis:

(79) $$\begin{array}{c} {\langle {\tilde{I}}_{D1X}\rangle }_{\left|\right|}\left(\beta ,\kappa ;\theta_{1}=\frac{\pi }{4};\Omega_{1},\Omega_{2},\Omega_{3}\right)\\ =\frac{{\langle R_{1}\rangle }_{\left|\right|}\left(\Omega_{1}\right)+{\langle R_{2}\rangle }_{\left|\right|}\left(\Omega_{2}\right)}{2}+{\left(-1\right)}^{n(X)}{\langle D\rangle }_{\left|\right|}\left(\Omega_{1},\Omega_{2},\Omega_{3}\right)\cos\left(2\beta \right)\cos\kappa , \end{array}$$

(80) $${\langle {\tilde{I}}_{D2X}\rangle }_{\left|\right|}\left(\theta_{2}=\frac{\pi }{4};\Omega_{1},\Omega_{2},\Omega_{3}\right)=\frac{{\langle R_{1}\rangle }_{\left|\right|}\left(\Omega_{1}\right)+{\langle R_{2}\rangle }_{\left|\right|}\left(\Omega_{2}\right)}{2}+{\left(-1\right)}^{n(X)}{\langle D\rangle }_{\left|\right|}\left(\Omega_{1},\Omega_{2},\Omega_{3}\right),$$

where

(81) $${\langle R_{1}\rangle }_{\left|\right|}\left(\Omega_{1}\right)+{\langle R_{2}\rangle }_{\left|\right|}\left(\Omega_{2}\right)=2g|V|B^{2}\left[\cos\Omega_{1}+\cos\Omega_{2}\right]+4g^{2}{\left|V\right|}^{2}A^{\prime }.$$

Next, the following question will be addressed: which fixed values of $\Omega_{1}$, $\Omega_{2}$ and $\Omega_{3}$ could give rise to the same amount of information that the one given in BSM does? In other words, is it there the possibility to translate the information obtained through the Bell-state analysis to information related to the phases of the vacuum field entering the source? This question makes sense in the framework of the WRHP formalism, where photons are represented by wave packets that are generated in the crystal and propagate throughout the setup from the source to the detectors. Given that correlated detectors are characterised for having identical expressions for the signal intensities, the question of which is the property of the field directly related to a joint photo-detection event arises. From now on, the following hypothesis will be considered in the case of perfect detectors:

*For an ideal photodetector, DjX, a detection is produced in the situation in which the SIAM, ${\langle {\tilde{I}}_{DjX}\rangle }_{\left|\right|}$, takes its maximum value as a function of the vacuum phases. In this way, in two correlated detectors where a joint detection is produced, the SIAMs are maximum and equal.*

### 8.1. Vacuum Phases in the Rectilinear Analysis

In this situation, a detection is produced in D2V (D2H) in the case $\Omega_{1}=0$ ($\Omega_{2}=0$), so that (see Equations (69), (70), (77) and (78)):

(82) $${\langle R_{i}\rangle }_{||,max}={\langle R_{i}\rangle }_{\left|\right|}\left(\Omega_{i}=0\right)=2g|V|B^{2}+2g^{2}{\left|V\right|}^{2}A^{\prime }\;\;\;;\;\;\;i=\left\{1,2\right\},$$

(83) $${\langle {\tilde{I}}_{D2H}\rangle }_{||,max}\left(\theta_{2}=0\right)={\langle R_{2}\rangle }_{\left|\right|}\left(\Omega_{2}=0\right)=2g|V|B^{2}+2g^{2}{\left|V\right|}^{2}A^{\prime },$$

(84) $${\langle {\tilde{I}}_{D2V}\rangle }_{||,max}\left(\theta_{2}=0\right)={\langle R_{1}\rangle }_{\left|\right|}\left(\Omega_{1}=0\right)=2g|V|B^{2}+2g^{2}{\left|V\right|}^{2}A^{\prime }.$$

It is obvious that if a joint detection event is produced at two correlated detectors, then the other two detectors cannot be activated. Therefore, a new question must be addressed: which is the relationship between the absence of joint detection and the values taken by the vacuum phases? In principle, one could be tempted to state that the corresponding SIAMs should be minimal. In this situation, by using Equations (69), (70), (77) and (78), the corresponding value of $\Omega_{j}$ (j={1,2}), would be $\Omega_{j}=\pi$. Then, by substituting $\Omega_{i}=0$ and $\Omega_{j}=\pi$, i≠j, in Equation (71), a null value for ${\langle D\rangle }_{\left|\right|}$ would be obtained, giving rise to identical values of ${\langle {\tilde{I}}_{D1X}\rangle }_{\left|\right|}\left(\beta ,\kappa ;\theta_{1}=\pi /4\right)$ and ${\langle {\tilde{I}}_{D2X^{\prime }}\rangle }_{\left|\right|}\left(\theta_{2}=\pi /4\right)$, ∀ *X*, $X^{\prime }$∈{H,V} (see Equations (79) and (80)). In such a situation, the possibility of establishing a relationship between BSM and the values of the phases of the zeropoint field would be prevented.

From now on, the following conjecture will be considered for the characterisation of non detection events in the rectilinear analysis:

*The first order contribution to the SIAMs, corresponding to two non activated detectors, is zero.*

This happens for $\Omega_{i}=\pm \pi /2$, that is:

(85) $${\langle R_{i}\rangle }_{\left|\right|}\left(\Omega_{i}=\pm \frac{\pi }{2}\right)=2g^{2}{\left|V\right|}^{2}A^{\prime }\;\;\;;\;\;\;i=\left\{1,2\right\},$$

(86) $${\langle {\tilde{I}}_{D2H}\rangle }_{\left|\right|}\left(\theta_{2}=0;\Omega_{2}=\pm \frac{\pi }{2}\right)={\langle R_{2}\rangle }_{\left|\right|}\left(\Omega_{2}=\pm \frac{\pi }{2}\right)=2g^{2}{\left|V\right|}^{2}A^{\prime },$$

(87) $${\langle {\tilde{I}}_{D2V}\rangle }_{\left|\right|}\left(\theta_{2}=0;\Omega_{1}=\pm \frac{\pi }{2}\right)={\langle R_{1}\rangle }_{\left|\right|}\left(\Omega_{1}=\pm \frac{\pi }{2}\right)=2g^{2}{\left|V\right|}^{2}A^{\prime }.$$

By taking into consideration that in the case β=0 (β=π/2), which corresponds to the states $|\Psi^{\pm }\rangle$ ($|\Phi^{\pm }\rangle$), detectors corresponding to orthogonal (identical) polarisations are correlated, one of the following two couples $\left(\Omega_{1},\Omega_{2}\right)$ could be associated to every couple (β,κ) and activated detectors (D1X,D2Y): $\left(\Omega_{1}=0,\Omega_{2}=\pm \pi /2\right)$ and $(\Omega_{1}=\pm \pi /2,$$\Omega_{2}=0)$. Each of these two couples would identify a given sequence of joint detections for every state:

- For the states $|\Psi^{\pm }\rangle$ ($|\Phi^{\pm }\rangle$), the couple $\left(\Omega_{1}=0,\Omega_{2}=\pm \pi /2\right)$ would correspond to the joint detection (D1H,D2V)[(D1V,D2V)]. In both cases, the detector D2V is activated (see Equation (78)).
- The couple $\left(\Omega_{1}=\pm \pi /2,\Omega_{2}=0\right)$ would identify a joint detection (D1V,D2H) for $|\Psi^{\pm }\rangle$, and (D1H,D2H) for $|\Phi^{\pm }\rangle$. In both cases, a detection is produced at detector D2H (see Equation (77)).

In this analysis, the value of $\Omega_{3}$ remains completely unknown. It must be emphasised that the amount of information that a concrete joint detection event gives about the state is equal to the one corresponding to the couple $\left(\Omega_{1},\Omega_{2}\right)$, both being complementary. Table 1 illustrates the relationship between BSM in the rectilinear basis and the information provided by the vacuum phases.

### 8.2. Vacuum Phases in the Diagonal Analysis

From Equations (71), (79) and (80), the analysis of the maximum value of ${\langle {\tilde{I}}_{D1X}\rangle }_{\left|\right|}$ and ${\langle {\tilde{I}}_{D2X}\rangle }_{\left|\right|}$ depends on the behaviour of ${\langle D\rangle }_{\left|\right|}$ as a function of $\Omega_{3}$, for the possible couples $\left(\Omega_{1}=0,\Omega_{2}=\pm \pi /2\right)$ and $\left(\Omega_{1}=\pm \pi /2,\Omega_{2}=0\right)$. The function ${\langle D\rangle }_{\left|\right|}$ becomes:

(88) $$\begin{array}{c} {\langle D\rangle }_{\left|\right|}\left(\Omega_{1}=0,\Omega_{2}=\pm \frac{\pi }{2},\Omega_{3}\right)={\langle D\rangle }_{\left|\right|}\left(\Omega_{1}=\pm \frac{\pi }{2},\Omega_{2}=0,\Omega_{3}\right)\\ =g|V|B^{2}\left(1+g|V|\right)f_{\pm }\left(\Omega_{3}\right)\;\;\;;\;\;\;f_{\pm }\left(\Omega_{3}\right)=\cos\Omega_{3}\pm \sin\Omega_{3}. \end{array}$$

By substituting Equation (88) into Equations (79) and (80), and by taking into account that $\cos\Omega_{1}+\cos\Omega_{2}=1$ in Equation (81), we have:

(89) $$\begin{array}{c} {\langle {\tilde{I}}_{D1X}\rangle }_{\left|\right|}\left(\beta ,\kappa ;\theta_{1}=\frac{\pi }{4};\Omega_{3}\right)=g|V|B^{2}+2g^{2}{\left|V\right|}^{2}A^{\prime }\\ +{\left(-1\right)}^{n(X)}g|V|B^{2}\left(1+g|V|\right)f_{\pm }\left(\Omega_{3}\right)\cos\left(2\beta \right)\cos\kappa , \end{array}$$

(90) $${\langle {\tilde{I}}_{D2X}\rangle }_{\left|\right|}\left(\theta_{2}=\frac{\pi }{4};\Omega_{3}\right)=g|V|B^{2}+2g^{2}{\left|V\right|}^{2}A^{\prime }+{\left(-1\right)}^{n(X)}g|V|B^{2}\left(1+g|V|\right)f_{\pm }\left(\Omega_{3}\right).$$

The relative extreme of $f_{\pm }\left(\Omega_{3}\right)$ give the maximum and minimum values of ${\langle D\rangle }_{\left|\right|}$. Let us consider the following cases:

- *Case I*. Sign “+”, i.e., $\left(\Omega_{1}=0,\Omega_{2}=\pi /2\right)$ or $\left(\Omega_{1}=\pi /2,\Omega_{2}=0\right)$. In this situation:
  (91) $$f_{+,max}=f\left(\Omega_{3}=\frac{\pi }{4}\right)=\sqrt{2}\;\;\;\;;\;\;\;\;f_{+,min}=f\left(\Omega_{3}=\frac{-3\pi }{4}\right)=-\sqrt{2}.$$
- *Case II*. Sign “−”, i.e., $\left(\Omega_{1}=0,\Omega_{2}=-\pi /2\right)$ or $\left(\Omega_{1}=-\pi /2,\Omega_{2}=0\right)$. In this case,
  (92) $$f_{-,max}=f\left(\Omega_{3}=\frac{-\pi }{4}\right)=\sqrt{2}\;\;\;\;;\;\;\;\;f_{-,min}=f\left(\Omega_{3}=\frac{3\pi }{4}\right)=-\sqrt{2}.$$

The maximum value of the SIAM, corresponding to a joint detection event, is given by:

(93) $${\langle {\tilde{I}}_{DjX}\rangle }_{||,max}\left(\theta_{j}=\frac{\pi }{4}\right)=g|V|B^{2}+2g^{2}{\left|V\right|}^{2}A^{\prime }+g|V|B^{2}\left(1+g|V|\right)\sqrt{2}\;\;\;;\;\;\;j=\left\{1,2\right\},X=\left\{H,V\right\}.$$

On the other hand, in the diagonal analysis, the SIAM takes its minimal value for two detectors where no joint detection is produced:

(94) $${\langle {\tilde{I}}_{DjX}\rangle }_{||,min}\left(\theta_{j}=\frac{\pi }{4}\right)=g|V|B^{2}+2g^{2}{\left|V\right|}^{2}A^{\prime }-g|V|B^{2}\left(1+g|V|\right)\sqrt{2}\;\;\;;\;\;\;j=\left\{1,2\right\},X=\left\{H,V\right\}.$$

By observing Equation (90), and taking into consideration that n(H)=1 and n(V)=2, a detection event is produced at the detector D2V (D2H) if $f_{\pm }=f\left(\Omega_{3}\right)$ takes its maximum (minimum) value, which gives rise to a maximum value for the SIAM ${\langle {\tilde{I}}_{D2V}\rangle }_{\left|\right|}$ (${\langle {\tilde{I}}_{D2H}\rangle }_{\left|\right|}$). Given that the signal intensities for two correlated detectors are equal, and by using the results described in Equations (67) and (68), the relationship between BSM in the diagonal basis and the information concerning $\Omega_{3}$, conditioned by the couples $\left(\Omega_{1}=0,\Omega_{2}=\pm \pi /2\right)$ or $\left(\Omega_{1}=\pm \pi /2,\Omega_{2}=0\right)$ obtained in the rectilinear analysis, is illustrated in Table 2.

By using the information provided by Table 1 and Table 2, the distinction of a given Bell state through two consecutive experiments, one of them in the rectilinear basis and the other in the diagonal one, can be associated to one of the four triple lists, (0,±π/2,∓3π/4), (0,±π/2,±π/4), (±π/2,0,∓3π/4), and (±π/2,0,±π/4), depending on the sequence of activated detectors for each analysis (see Table 3). The amount of obtained information, either via Bell-state distinction or through the extraction of phase information from the vacuum field, is the same, independently of the number of performed experiments, as shown by Table 1, Table 2 and Table 3.

## 9. Discussion and Conclusions

In this paper, the WRHP formalism of quantum optics has been applied to a Bell-type experiment, in which the two photons, entangled in any of the four polarisation Bell states (represented by the preparation parameters β and κ) do not interact at the Bell-state analyser. The signal intensities at the detectors, i.e., the intensities above the pure zeropoint intensity (see Equation (30)), have been calculated as functions of the phases and modules of the four zeropoint amplitudes that couple with the laser field, and the measuring parameters (represented by the angles $\theta_{1}$ and $\theta_{2}$). This calculation (see Equations (45)–(51)) constitutes a generalisation of a previous one in the rectilinear basis [1]. The relationship between the Bell-state analysis and phase information in the beam-splitter-based BSM [21] will be investigated in a further work.

One of the main results of this paper has been derived in Section 7. The signal intensities, as functions of the phases and modules of the zeropoint amplitudes, are equal for two correlated detectors. This property allows for a characterisation in which detectors are correlated, without involving the calculation of the joint detection rates. Based on this result, in Section 8, a table of information has been generated concerning the values of the phases of the ZPF, $\Omega_{1}$, $\Omega_{2}$ and $\Omega_{3}$ (see Equations (35)–(37)), in the complete analysis of Bell states through two consecutive experiments, one of them in the rectilinear basis and the other in the diagonal one (see Table 3). This table gives the same amount of information, but is complementary to the one provided by the usual analysis that relates Bell states and which-path information.

The generation of Table 3 implies the use of the SIAMs, i.e., the average of the signal intensities integrating over the modules of the zeropoint amplitudes, and the consideration of a conjecture concerning the nexus between the joint photodetection at two correlated detectors and the maximum value taken for the SIAM as a function of the vacuum phases. In addition, the hypothesis that non detection at the other two detectors is related to the null value of the first order term in the SIAM in the rectilinear analysis, leads to the couples of values $\left(\Omega_{1}=\pm \pi /2,\Omega_{2}=0\right)$ and $\left(\Omega_{1}=0,\Omega_{2}=\pm \pi /2\right)$ that are applied further in the diagonal analysis. In this sense, BSM can be observed as a way of extracting phase information from the ZPF. The knowledge of the pair of activated detectors for each couple of the preparation parameters, (β,κ), is directly related to the values taken by the vacuum phases at the source of entanglement. The visible presence of the ZPF in an optical quantum communication has been previously emphasised via the use of the WRHP formalism [1,4], and similar approaches are leading to the consideration of a real zeropoint field [25,26].

The consideration of fixed values for the vacuum phases leading to detection seems to contradict the consideration of the randomness of the zeropoint amplitudes in the prediction of the single and joint detection rates (see Equation (6)). From our point of view, the same controversy appears when the detection is related to which-path information, in the sense that photons cannot be associated with particles that travel from the source to the detectors through the setup [27,28,29,30].

In the rectilinear analysis, the relationship between $\Omega_{1}$ and $\Omega_{2}$, $\Omega_{1}+\Omega_{2}=\pm \pi /2$, is obtained by considering that if the SIAM is maximal in two correlated detectors, the absence of a joint detection event at the other couple of detectors reveals that the first-order term of the corresponding signal intensity must be zero. Although this relationship could, in principle, be considered as artificial, it is supported by a recent work of Jung [31]. Moreover, and this should be studied in a further work, our conjecture is that the results concerning the calculation of single and joint detection rates in the WRHP formalism are invariant under the exchange $\Omega_{2}\to \pm \pi /2-\Omega_{1}$. In our opinion, the relationship proposed in [31] between the phases of the two strongly correlated wave-packets emitted by the crystal has its origin in the ZPF amplitudes at the source of entanglement, as pointed out in this work.

From Equations (69)–(71), and (48)–(51), it can be observed that the values of $\Omega_{1}$, $\Omega_{2}$ and $\Omega_{3}$ leading to a maximum value of the SIAMs, for any values of the measuring ($\theta_{1}$, $\theta_{2}$) and preparation (β, κ) parameters, are dependent on the concrete experimental context. In this way, although the vacuum phases are intrinsically stochastic, each experiment selects fixed values of $\Omega_{i}$ compatible with the results (joint detection). In this sense, in Equations (83), (84), (86), (87), (93) and (94), the maximum and minimum values of the SIAMs in the rectilinear and diagonal analyses, respectively, show the following counterintuitive results: (a) the corresponding values of the maximal SIAMs are different; (b) the values of the corresponding intensities to non activated detectors are different in both basis; (c) the minimal values of the SIAMs in the rectilinear (diagonal) analysis are positive (negative), which can be deduced by substituting the relationship between $B^{2}/A^{\prime }$ (see Equation (74)) into Equations (93) and (94). From our point of view, these results are consistent with the quantum contextuality [32,33,34,35,36], but this subject deserves further study.

## Figures and Tables

**Table 1:** Partial Bell-state analysis and phase information in the rectilinear basis ($\theta_{1}=\theta_{2}=0$).

Class	States	Activated Detectors	Phase Information ($\Omega_{1}$, $\Omega_{2}$)
1	$\{|\Psi^{+}\rangle ,|\Psi^{-}\rangle \}$	(D1H, D2V)	(0, $\pm \frac{\pi }{2}$)
		(D1V, D2H)	($\pm \frac{\pi }{2},0$)
2	$\{|\Phi^{+}\rangle ,|\Phi^{-}\rangle \}$	(D1H, D2H)	($\pm \frac{\pi }{2},0$)
		(D1V, D2V)	($0,\pm \frac{\pi }{2}$)

**Table 2:** Partial Bell-state analysis and phase information in the diagonal basis ($\theta_{1}=\theta_{2}=\pi /4$).

Class	States	Activated Detectors	Phase Information $\Omega_{3}$
1	$\{|\Psi^{-}\rangle ,|\Phi^{-}\rangle \}$	(D1H, D2V)	$\pm \frac{\pi }{4}$
		(D1V, D2H)	$\mp \frac{3\pi }{4}$
2	$\{|\Psi^{+}\rangle ,|\Phi^{+}\rangle \}$	(D1H, D2H)	$\mp \frac{3\pi }{4}$
		(D1V, D2V)	$\pm \frac{\pi }{4}$

**Table 3:** Complete Bell-state analysis and phase information by considering two consecutive experiments.

State	Rectilinear Analysis	Diagonal Analysis	Phase Information ($\Omega_{1}$, $\Omega_{2}$, $\Omega_{3}$)
$|\Psi^{+}\rangle$	(D1H, D2V)	(D1H, D2H)	($0,\pm \frac{\pi }{2},\mp \frac{3\pi }{4}$)
	(D1H, D2V)	(D1V, D2V)	($0,\pm \frac{\pi }{2},\pm \frac{\pi }{4}$)
	(D1V, D2H)	(D1H, D2H)	($\pm \frac{\pi }{2},0,\mp \frac{3\pi }{4}$)
	(D1V, D2H)	(D1V, D2V)	($\pm \frac{\pi }{2},0,\pm \frac{\pi }{4}$)
$|\Psi^{-}\rangle$	(D1H, D2V)	(D1H, D2H)	($0,\pm \frac{\pi }{2},\mp \frac{3\pi }{4}$)
	(D1H, D2V)	(D1V, D2V)	($0,\pm \frac{\pi }{2},\pm \frac{\pi }{4}$)
	(D1V, D2H)	(D1H, D2H)	($\pm \frac{\pi }{2},0,\mp \frac{3\pi }{4}$)
	(D1V, D2H)	(D1V, D2V)	($\pm \frac{\pi }{2},0,\pm \frac{\pi }{4}$)
$|\Phi^{+}\rangle$	(D1H, D2H)	(D1H, D2V)	($\pm \frac{\pi }{2},0,\pm \frac{\pi }{4}$)
	(D1H, D2H)	(D1V, D2H)	($\pm \frac{\pi }{2},0,\mp \frac{3\pi }{4}$)
	(D1V, D2V)	(D1H, D2V)	($0,\pm \frac{\pi }{2},\pm \frac{\pi }{4}$)
	(D1V, D2V)	(D1V, D2H)	($0,\pm \frac{\pi }{2},\mp \frac{3\pi }{4}$)
$|\Phi^{-}\rangle$	(D1H, D2H)	(D1H, D2V)	($\pm \frac{\pi }{2},0,\pm \frac{\pi }{4}$)
	(D1H, D2H)	(D1V, D2H)	($\pm \frac{\pi }{2},0,\mp \frac{3\pi }{4}$)
	(D1V, D2V)	(D1H, D2V)	($0,\pm \frac{\pi }{2},\pm \frac{\pi }{4}$)
	(D1V, D2V)	(D1V, D2H)	($0,\pm \frac{\pi }{2},\mp \frac{3\pi }{4}$)

## Data Availability

Not applicable.

## Abbreviations

The following abbreviations are used in this manuscript:

ZPF	Zeropoint field
PDC	Parametric down-conversion
WRHP	Wigner representation in the Heisenberg picture
BSM	Bell-state measurement
LELM	Linear evolution and local measurement
PBS	Polarizing beam-splitter
SIAM	Signal intensity averaged in the modules of the vacuum amplitudes.
